# Fate and Transport of Shale-derived, Biogenic Methane

**DOI:** 10.1038/s41598-017-05103-8

**Published:** 2017-07-07

**Authors:** M. Jim Hendry, Erin E. Schmeling, S. Lee Barbour, M. Huang, Scott O. C. Mundle

**Affiliations:** 1 Department of Geological Sciences, University of Saskatchewan, 114 Science Place, Saskatoon, SK S7N 5E2 Canada; 2 Department of Civil, Geological and Environmental Engineering, University of Saskatchewan, 57 Campus Dr., Saskatoon, SK S7N 5A9 Canada; 3 Great Lakes Institute for Environmental Research, University of Windsor, 401 Sunset Ave., Windsor, ON N9B 3P4 Canada

## Abstract

Natural gas extraction from unconventional shale gas reservoirs is the subject of considerable public debate, with a key concern being the impact of leaking fugitive natural gases on shallow potable groundwater resources. Baseline data regarding the distribution, fate, and transport of these gases and their isotopes through natural formations prior to development are lacking. Here, we define the migration and fate of CH_4_ and δ^13^C-CH_4_ from an early-generation bacterial gas play in the Cretaceous of the Williston Basin, Canada to the water table. Our results show the CH_4_ is generated at depth and diffuses as a conservative species through the overlying shale. We also show that the diffusive fractionation of δ^13^C-CH_4_ (following glaciation) can complicate fugitive gas interpretations. The sensitivity of the δ^13^C-CH_4_ profile to glacial timing suggests it may be a valuable tracer for characterizing the timing of geologic changes that control transport of CH_4_ (and other solutes) and distinguishing between CH_4_ that rapidly migrates upward through a well annulus or other conduit and CH_4_ that diffuses upwards naturally. Results of this study were used to provide recommendations for designing baseline investigations.

## Introduction

Global natural gas reserves in organic-rich, low-permeability shales are estimated at 716 trillion m^3^ ^1^. Natural gas extracted from these unconventional shale gas reservoirs is an important energy resource being developed in several countries and is under consideration in several others^2^. However, animated public debate continues over possible environmental and human impacts^3^, in particular the impact of leaking fugitive natural gases (*i*.*e*., methane, C_1_; ethane, C_2_; propane, C_3_; butane, C_4_; and pentane, C_5_) and fluids from the development of unconventional gas plays on shallow potable groundwater resources^4–6^. Evaluation of these impacts relies on baseline data regarding the distribution, fate, and transport of these gases and their isotopes through natural formations prior to development. Such data are lacking, both in regard to detailed site-specific cross-formational distributions of organic gases as well as distributions within different geographic areas^4, 7^. Here, we define the migration and fate of CH_4_, the dominant unconventional gas, and δ^13^C-CH_4_ from an early-generation bacterial gas play in the Cretaceous of the Williston Basin (WB), Canada^8^ to the water table and provide guidance on designing baseline investigations.

**Figure 1:**
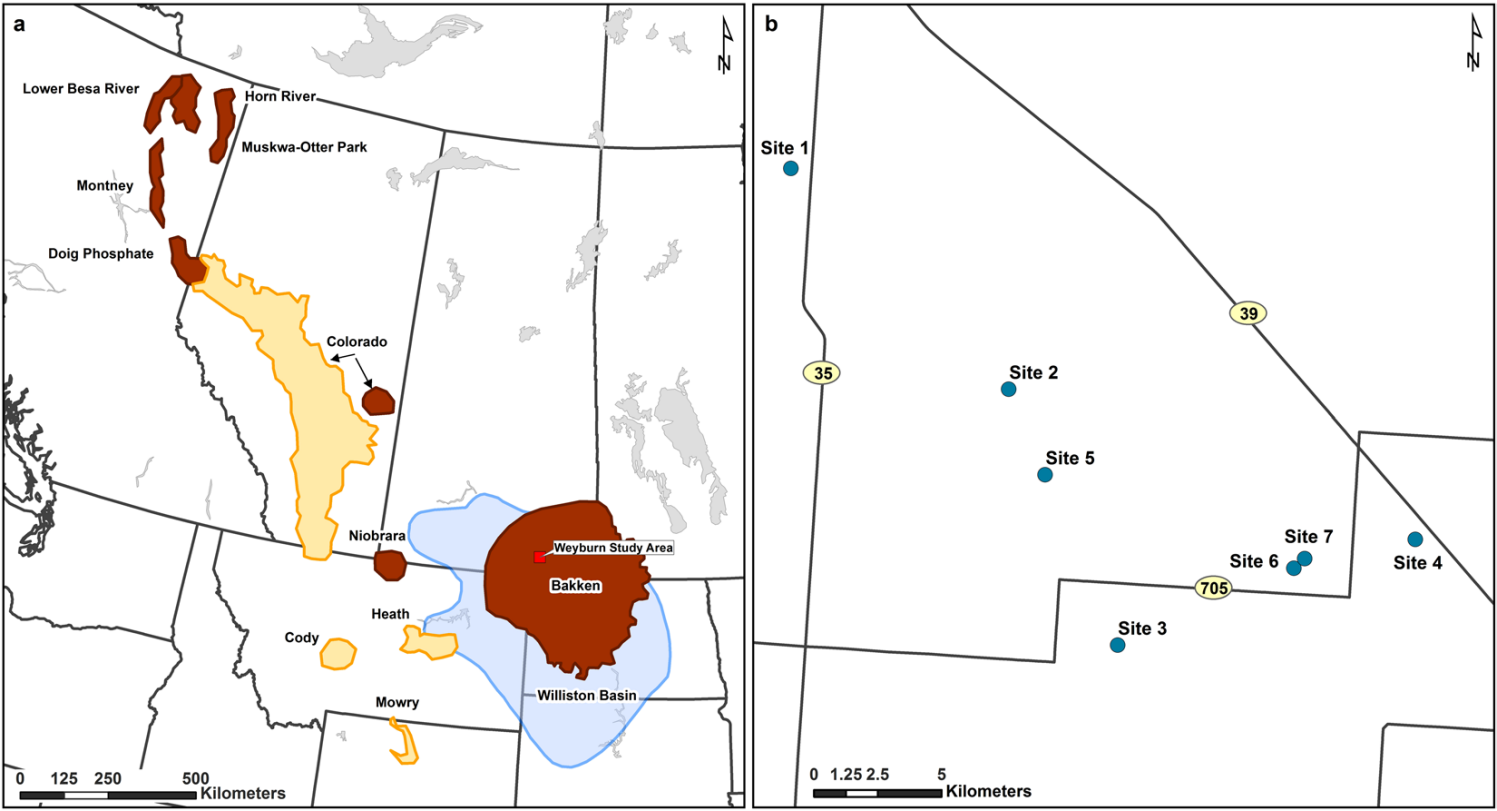
Location map. (**a**) Extent of the Western Canadian Sedimentary Basin (yellow), Williston Basin (blue)^43^, and Bakken shale play (orange)^3^ in northwestern USA and western Canada, and location of study area. (**b**) Location of Sites 1–4^15^, Site 5^32^, and Sites 6 and 7. Highways are shown as solid black lines in (**b**). Adapted with permission from ref. 3 and ref. 54. Copyright 2014 American Chemical Society and 2012 Springer International, respectively. Map was created with ArcMap version 10.5 (http://esri.ca/en/products/whats-new-in-arcgis-105).

The WB is an important reservoir of unconventional energy in North America underlying 250,000 km^2^ of Montana and North Dakota in the USA and Saskatchewan and Manitoba in Canada (Fig. 1a). The dominant unconventional energy play in the WB is in the Bakken Formation^9, 10^ (Fig. 1a) and is characterized as thermogenic in origin^11^. Unconventional gas-producing zones also exist near the base of thick, laterally extensive, younger Cretaceous-age shales in the WB and Western Canadian Sedimentary Basin (Fig. 1a). We collected detailed gas molecular and isotopic composition profiles from the overlying glacial deposits, that are widespread throughout the WCSB, with thicknesses up to 300 m^12^, through the Cretaceous shale at two sites, Sites 2 and 5, to depths of 150 and 200 m below ground surface (BG), respectively. We also collected the same data from the water table through the gas source zone at two additional sites, Sites 6 and 7, to 971 m BG. All sites were located near Weyburn, Saskatchewan (lat: 49.6633; long: 103.8533) (Fig. 1b).

**Figure 2:**
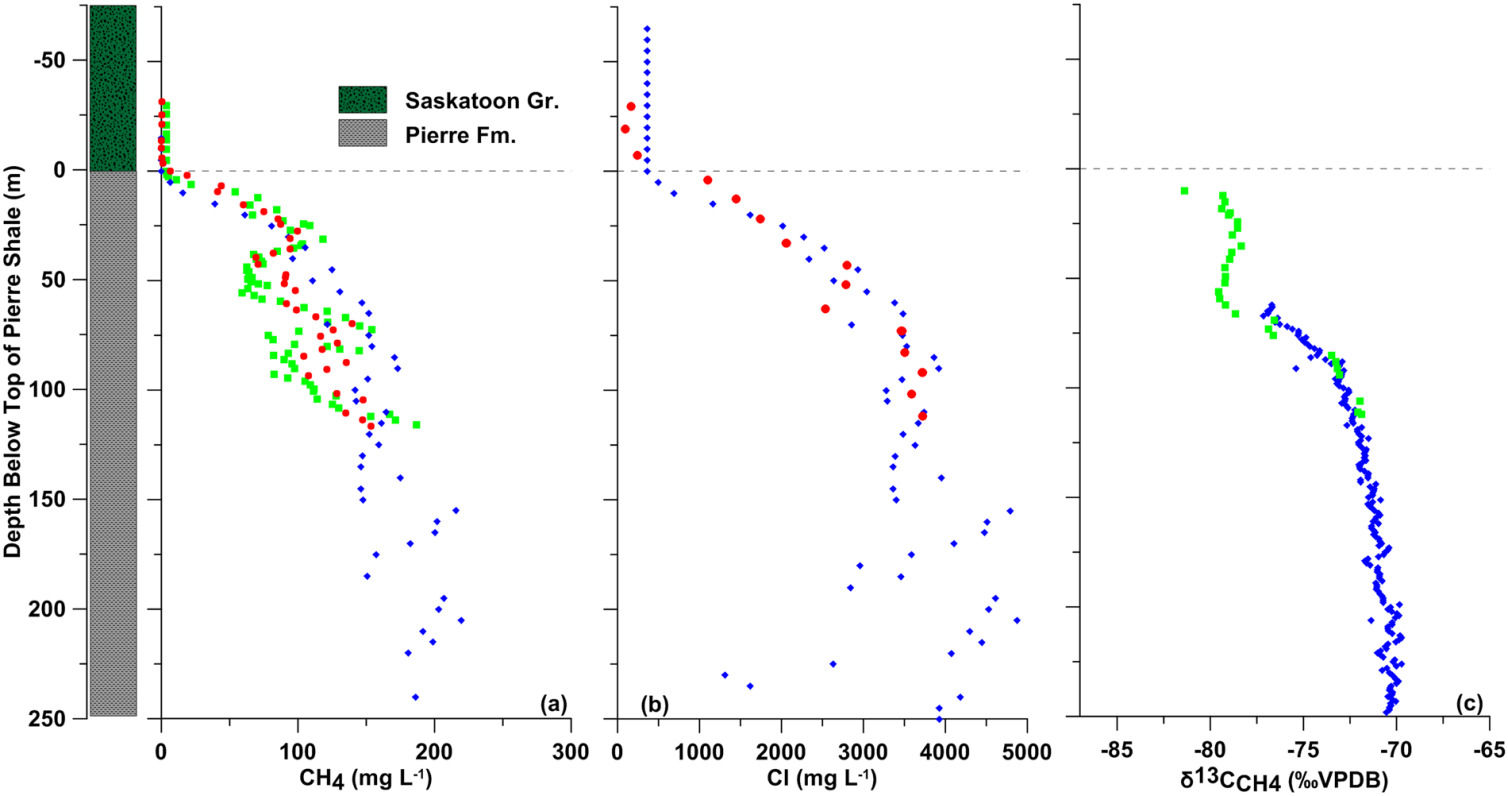
Geology (Site 2) and dissolved CH_4_ concentrations (**a**), Cl^−^ concentrations (**b**), and δ^13^C-CH_4_ values (**c**) vs. depth below the top of the Pierre Shale at Sites 2 and 6. The Isojar^®^ data from Site 2 are shown as solid red dots, and mud gas data from Sites 2 and 6 as solid green squares and solid blue triangles, respectively.

**Figure 3:**
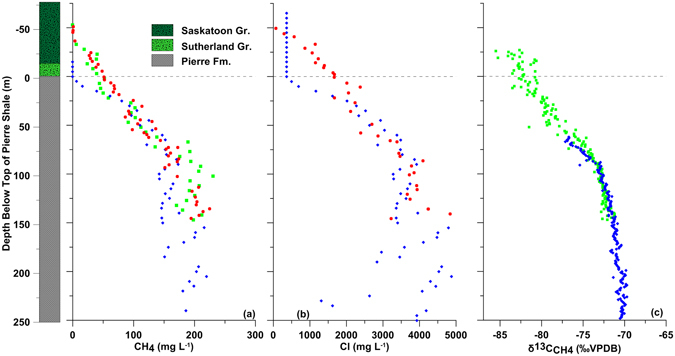
Geology (Site 5) and dissolved CH_4_ concentrations (**a**), Cl^−^ concentrations (**b**), and δ^13^C-CH_4_ values (**c**) vs. depth below the top of the Pierre Shale at Site 5. Site 6 data from Fig. 2 are added in this figure to show the different CH_4_ and Cl^−^ profiles at Site 5 versus Sites 2 and 6. The Isojar^®^ data from Site 5 are shown as solid red dots, and mud gas data from Sites 5 and 6 as solid green squares and solid blue triangles, respectively.

**Figure 4:**
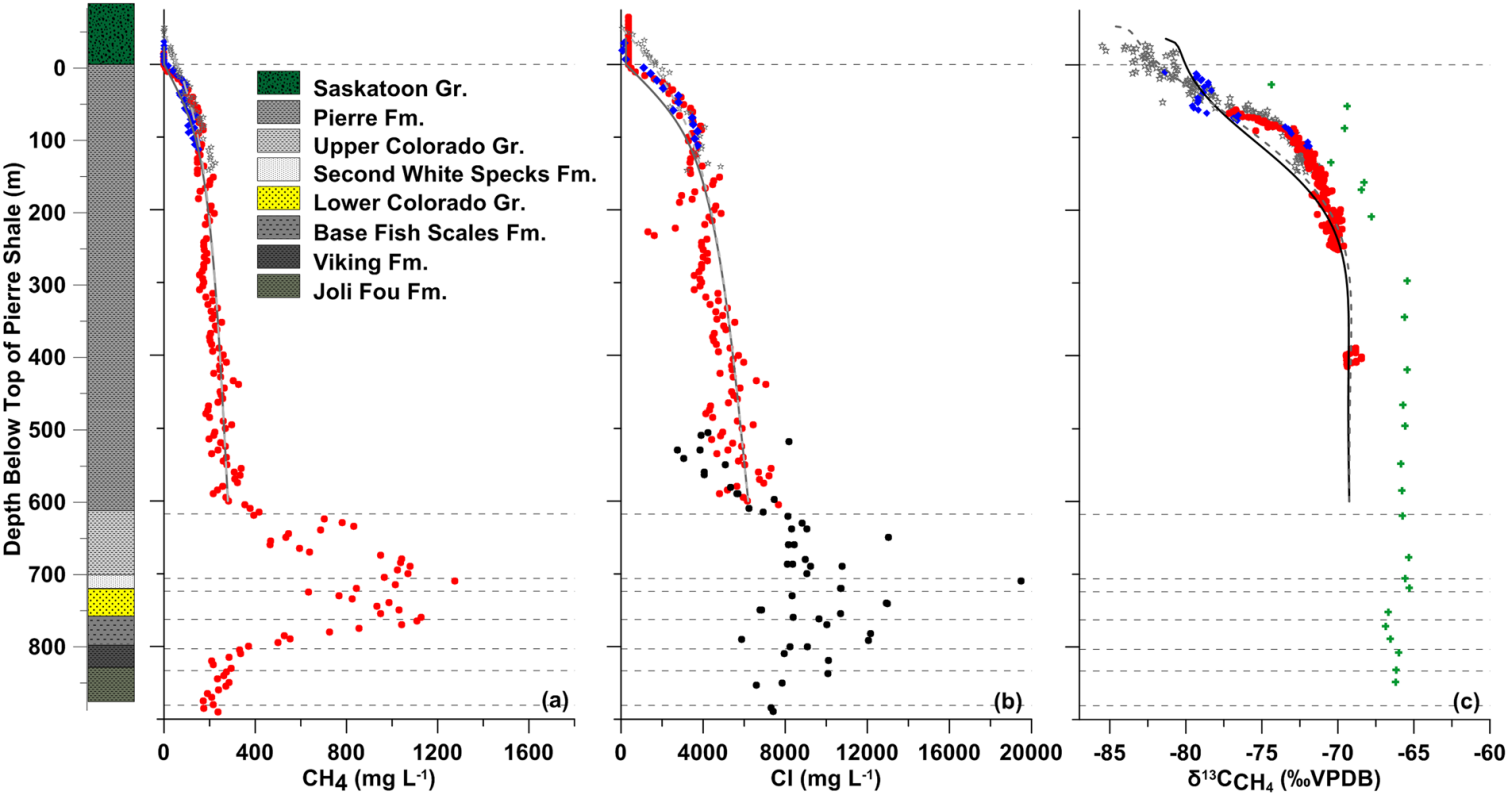
Geology and dissolved CH_4_ concentrations (**a**), Cl^−^ concentrations (**b**), and δ^13^C-CH_4_ values (**c**) through the Quaternary deposits and Cretaceous shales at Sites 2, 5, 6, and 7. In (**a**) and (**b**), Isojar^®^ data from Sites 2 and 5 are shown as solid blue diamonds and grey stars, respectively, and calibrated mud gas data from Sites 2, 5, and 6 as dashed black line, solid black line, and solid red circles, respectively. Measured Cl^−^ concentrations from the base of the Pierre Fm to the Joli Fou Fm from a drill site located 250 km SE of the study area^22^ are presented as solid black squares in (**b**). In (**c**), IsoTube® data from Sites 2 and 7 are presented as solid blue diamonds and green crosses, respectively, and mud gas data from Sites 5 and 6 as solid pink inverted triangles and solid red circles, respectively. The best-fit 1-D diffusive modeling results (1.6 Ma and 0.3 Ma for Sutherland and Saskatoon Group tills, respectively) are presented as solid lines (Sites 2 and 6) and dashed lines (Site 5) in (**a**), (**b**), and (**c**).

Overall, the geology across all sites is consistent (Figs 2–4). The upper 35 to 83 m consists of clay-rich, Quaternary-aged glacial sediments of the Saskatoon Group (0.01–0.6 Ma BP) and Sutherland Group (0.6–1.6 Ma BP)^13^. The surficial 8–10 m of till is brown (oxidized) and visibly fractured and the underlying till and bedrock is dark grey (anoxic) and nonfractured. The water table is located within the oxidized till zone (~6.4 and ~7.3 m BG at Sites 2 and 5, respectively). Cretaceous shale formations disconformably underlie the tills. The thick Pierre Shale Fm (~650 m thick; 72.1–83.6 Ma BP) directly underlies the tills. It consists of non-calcareous, grey to dark grey, hard, high plasticity silt and clay^14^. The Mannville Gr (~115 m thick; 100.5–145 Ma BP), located at the base of the Cretaceous, is a regionally extensive, sand-dominated aquifer and defines the base of our investigation.

We measured similar CH_4_ concentration-depth profiles below the water table at Sites 2, 5, and 6 (Figs 2–4). The upper portion of the CH_4_ profiles are concave downwards and increasing with depth from low (typically < 1.0 mg L^−1^) above the till-shale contact at Sites 2 and 6 or slightly below the anoxic-oxic till contact (12 m BG) at Site 5 to about 225 mg L^−1^ at 200 m below the top of the Pierre Shale Fm (BTS). The concentrations increase in a linear manner (y = −2.6x–220; n = 106; R^2^ = 0.66) from 250 m BTS to approximately 300 mg L^−1^ near the base of the Pierre Shale Fm (600 m BTS). The concentrations increase rapidly to > 1000 mg L^−1^ across the Colorado to the Base Fish Scales Fm (600–800 m BTS) with maximum concentrations in the Second White Specks and Lower Colorado Sand Fms (707–763 m BTS) where the hydrocarbons are generated (Fig. 4). Below the Base Fish Scales Fm, the concentrations decrease through the Viking and Joli Fou Fms to 236 mg L^−1^ at the top of the Mannville Gr (881 m BTS). Although the datasets are limited in areal extent, the consistency between the CH_4_ depth profiles from all sites and the fact we observe elevated CH_4_ concentrations in the Second White Specks Fm at a site located 250 km NE of the study area^15^ suggest our data represent general CH_4_ conditions in the Cretaceous shales across the WB.

Concentrations of CH_4_ through the Quaternary and shale deposits are below the solubility limit (Fig. S1) as estimated from *in vitro* gas concentrations corrected for *in situ* temperatures and pressures. From the Second White Specks Fm to the Base Fish Scales Fm, however, CH_4_ concentrations are equal to or exceed the estimated solubility limit suggesting the presence of free CH_4_ gas in these formations. This determination is supported by the presence of seismic reflectors believed to be associated with free gas in the same formations at other sites in the WB (pers. comm. *D Gendzwill*; pers. comm. *J*. *Szmigielski*). The Second White Specks Fm is a known early-generation biogenic gas reservoir in Cretaceous sediments of Western Canada^8^.

The dissolved chloride (Cl^−^) concentration-depth profiles at Sites 2 and 5 (Figs 2 and 3) positively correlate with the CH_4_ data; cross-plotted CH_4_ vs. Cl^−^ concentrations yield strong linear relationships (y = 0.04x + 3.4, R^2^ = 0.90, n = 15 and y = 0.06x + −28.5, R^2^ = 0.89, n = 35, respectively; Fig. S2. Given that Cl^−^ is a conservative species, these strong linear relationships suggest, on the whole, the same transport mechanism is operative for both Cl^−^ and CH_4_ with no indication of losses of CH_4_ from biogeochemical activity (i.e., no production or removal) in the CH_4_ concentration-depth profiles in the till and shale. The lack of sorption-desorption reactions for CH_4_ is supported by laboratory experiments and consistent with low concentrations of total organic carbon in the Pierre Shale (~0.8% wt)^16^.

The strong linear correlations over the entire data sets, however, do not apply at the upper boundaries for Cl^−^ and CH_4_ profiles at all sites (Fig. S2). We compared the upper boundaries for the Cl^−^ and CH_4_ profiles at Sites 2 and 5 to identify the controls on CH_4_ depletion at these locations (by either a biological or physical mass loss process). The upper boundaries for both the increasing Cl^−^ and CH_4_ depth profiles at Site 2 are at the till-shale interface (about 31 m BG). That both the Cl^−^ (240 mg/L) and CH_4_ (1.3 mg/L) concentrations were low and constant at and above this depth suggests the dominant control on both solutes at Site 2 is flushing/dilution by lateral water migration, likely through more permeable sediments in the glacial till^16^. In contrast to Site 2, the upper boundaries for Cl^−^ (450 mg/L) and CH_4_ (3.4 mg/L) at Site 5 are located at different depths in the till. The upper boundary for the Cl^−^ profile is located at the base of the oxidized, fractured till zone (about 4.5 m BG) whereas the upper boundary for the CH_4_ profile is in the underlying anoxic till (about 12 m BG). Unlike at Site 2, the extension of both Cl^−^ and CH_4_ profiles well into the till at Site 5 is attributed to the lack of permeable zones in the till above the shale (*i*.*e*., no permeable layers were observed in continuous core samples from the till). The upward migration of solutes from the shale into the overlying till at Site 5 is observed in other tills and also attributed to a lack of permeable zones^17, 18^. The presence of the upper boundary for the Cl^−^ profile in the oxidized till at Site 5 is attributed to the presence of fractures (higher *K*) in this zone that allows solutes to be transported laterally with flowing groundwater^19^. In contrast, the presence of the upper CH_4_ boundary in the underlying anoxic till is attributed to CH_4_ oxidation associated with the reduction of SO_4_^2−^ generated in the oxidized till by the oxidation of reduced sulphur during the Altithermal Period, a mid-Holocene warming period between 9000 and 5000 years before present^20–22^.

Although the shallow data at Site 6 are limited, the trend in CH_4_ at the upper boundary is consistent with Site 2. The geologic log for Site 6 also shows the till has a high sand content, indicative of the presence of permeable sand layers. The fact that the upper boundaries for Cl^−^ and CH_4_ at Sites 3 and 4^16^, also located in our study area (Fig. 1b), coincide with the presence of a permeable zone at the top of the Pierre Shale Fm suggests that physical control (*i*.*e*., lateral flushing) may be more common at the upper boundary for CH_4_ than the biogeochemical reactions (*i*.*e*., methane oxidation) observed at Site 5.

The overall strong correlation between CH_4_ and Cl^−^ profiles at Sites 2 and 5 was used to estimate Cl^−^ concentrations through the Pierre Shale at Site 6. The good visual fits between the estimated Cl^−^ profile and those measured on squeezed samples from Sites 2 and 5 (Figs 2b and 3b) and the estimated Cl^−^ concentrations being consistent with those measured through the lower 275 m of Cretaceous shales at a site ~250 km SE of the study area^23^ (Fig. 4b) provide confidence in the calculated Cl^−^ profile for Site 6. As was the case for the CH_4_ profiles, the Cl^−^ profiles exhibit well-defined, slight curvilinear (concave down) depth trends to about 200 m BTS after which they increase in a linear manner from 250 m BTS to near the base of the Pierre Shale Fm (600 m BTS) (Fig. 4b). Based on Cl^−^ concentration profiles with depth through the WB, the source of Cl^−^ in the Cretaceous shales of the WB was determined to be the Prairie Evaporite Fm, located about 1800 m BG in our study area^23^.

The shapes of the CH_4_ and Cl^−^ concentration profiles suggest the dominant transport mechanism for these species is diffusion. Diffusive transport in the Pierre Shale Fm and anoxic tills is supported by low measured hydraulic conductivity (*K*) values of Cretaceous shale (10^−12^–10^−13^ m/s)^24^ and anoxic tills (10^−10^–10^−11^ m/s)^25, 26^ in the WB. The dominance of diffusive transport in low *K* argillaceous sediments is consistent with findings from other argillaceous systems in the WB or proximal to it^15, 23, 27, 28^ and in other areas of the world^29–33^.

We modeled the CH_4_ and Cl^−^ concentration profiles in the Pierre Shale Fm and Quaternary tills at Sites 2 and 5 assuming diffusion to be the dominant solute transport mechanism. Results of modelled scenarios are summarized in Figs 4 and S6 to S11 and the model parameters used are summarized in Table S1. The curvilinear profiles in the upper 200 m of the profiles developed in response to assumed abrupt changes in the upper boundary condition. These abrupt changes are attributed to the effects of glaciation^17, 27^. The good fits between the modeling results and measured data for time frames consistent with known ages of shale and glacial deposition confirm that long-term upward diffusive transport over many millions of years can explain the current measured Cl^−^ and CH_4_ profiles. Although the times (*i*.*e*., onset of Sutherland Gr. glaciation at 1.6 Ma and onset of Saskatoon Gr. glaciation between 0.1 and 0.6 Ma) used to simulate the measured profiles (especially the upper 200 m of Pierre Shale Fm) are consistent with the timing of the glaciations, the results cannot be considered unique because they are based on a limited understanding of the geologic history of the shale and tills. However, as we show below, the estimates of timing can also be constrained by simulating the δ^13^C-CH_4_ profiles.

The δ^13^C-CH_4_ depth profiles at all sites exhibit a well-defined curvilinear enrichment in ^13^C with increasing depth to about 250 m BG (Figs 2–4). Over this depth, the δ^13^C-CH_4_ values increase from about −85‰ to about −70‰. Below about 250 m BG depth, the δ^13^C-CH_4_ values are constant, ranging from −65.3 to −68.5‰ (depending on sampling method).

The δ^13^C-CH_4_ values (−72‰ to −64‰) and lower concentration ratios of CH_4_ to the heavier hydrocarbons (C_1_/C_2+_; >10; Fig. S12) for all samples collected in this study are consistent with early-generation bacterial gas^8^. Late-generation biogenic gases are often thought to be ^13^C-depleted relative to these values and have concentration ratios of CH_4_ to the heavier hydrocarbons (C_1_/C_2+_; >1000)^34–36^.

Well-defined changes in the δ^13^C-CH_4_ values with depth such as those measured across the Pierre Shale are not documented in the literature. A similar isotopic shift to that observed in the upper 250 m of shale was, however, measured on mud gas samples at a site near Brooks, Alberta (600 km NW of our study area)^37^. In that study, the δ^13^C-CH_4_ values increased from −80‰ at depths <50 m BG to −62‰ at about 150 m BG and remained constant at this value to 530 m BG. The similarity between the Brooks profile and our profiles, and given the 600-km distance between these sites, suggests the δ^13^C-CH_4_ profiles in our study area represent conditions across the WB. Some authors suggest changes in δ^13^C-CH_4_ values can be caused by CH_4_ transport in the subsurface^16, 38–40^ while others discount the effects of δ^13^C-CH_4_ migration on δ^13^C values^41^.

The constant δ^13^C-CH_4_ values measured below 250 m BG are consistent with near steady-state CH_4_ transport through the shale prior to glaciation (as demonstrated in the modelling). An abrupt change in the upper CH_4_ boundary condition, such as glaciation, would trigger transient diffusion that would cause diffusive isotopic fractionation. The isotopic shift in the δ^13^C-CH_4_ profiles to 250 m BG is consistent with experimental results that show diffusing CH_4_ has a significantly ^13^C-depleted δ^13^C value than its source^38^. Because the CH_4_ and Cl^−^ profiles in our study can be explained via diffusive transport, we assessed whether diffusive transport could produce the measured δ^13^C-CH_4_ profiles by conducting diffusive transport modeling of the δ^13^C-CH_4_ depth profiles through the shale at Sites 2 and 5. The simulation results using a consistent set of transport conditions to those used in the CH_4_ simulations yield reasonable fits to the measured data (Fig. 4) and suggest that isotopic fractionation as a result of differences in the diffusion of ^12^C and ^13^C in the CH_4_ can explain the measured δ^13^C-CH_4_ profile. As was the case for the CH_4_ and Cl^−^ simulations, the δ^13^C-CH_4_ simulations are non-unique.

Based on high-resolution profiling, we gained an understanding of the origin, fate, and transport of CH_4_ in the WB. Specifically, the study provided insights into gas migration processes, CH_4_ source area delineation and methods to define upper boundary controls. Findings show the source of the CH_4_ and δ^13^C-CH_4_ in the shallow groundwater is an early-generation bacterial gas play located in the Lower Colorado sand and Second White Specks Fms. This CH_4_ migrates upward from its source area through the overlying shale and into the shallow subsurface via non-reactive diffusion over millions of years. Modelling the migration of the δ^13^C-CH_4_ profiles can be used to improve the estimates of timing obtained from modelling of the CH_4_ and Cl^−^ profiles. Our observations relating Cl^−^ and CH_4_ concentrations in the near surface environment can be used to define the controls on CH_4_ (*i*.*e*., flushing vs. microbiological). The Cl^−^ and CH_4_ concentrations data could also provide insight with respect to distinguishing recent CH_4_ production from gas migration in near surface aquifers. For example, if the concentrations of both CH_4_ and Cl^−^ in aquifer waters are elevated, the CH_4_ may be sourced deeper in the basin. However, if Cl^−^ concentrations are low relative to CH_4_ concentrations, CH_4_ is likely produced in the aquifer. The −15‰ diffusive shift in δ^13^C-CH_4_ over the upper 250 m of the WB can generate isotopic values comparable to those commonly associated with more recent methanogenic CH_4_ production in shallow potable aquifers (<−80‰)^22, 36, 42^; thus complicating our ability to distinguish CH_4_ produced in an aquifer from the impacts of CH_4_ that fractionated during migration from early-generation bacterial gas plays. This negative shift in carbon isotopes and co-migration of Cl^−^ allows us to distinguish CH_4_ contributions from pre-development CH_4_, recent microbial production, and migration from shallow bacterial gas plays (^13^C-depleted δ^13^C values relative to −65‰) from fugitive gases that rapidly migrate upward through a well annulus or other conduit originating from bacterial gas plays and deeper thermogenic oil-associated production zones in the WB (^13^C-enriched δ^13^C values relative to −65‰)^11, 42–44^. Although the large δ^13^C-CH_4_ fractionation resulting from diffusion can complicate identifying bacterial gas sources, our improved understanding of the regional dynamics of gas migration provides a new means to evaluate CH_4_ sources in potable groundwater resources in the WB and similar basins in western North America. Data generated in our study can also inform other valuable studies including those applying the model-derived upward flux of CH_4_ (6.6 × 10^−2^ mg m^−1^ d^−1^) to estimate long-term gas production from shallow biogenic gas plays.

The challenge to define the environmental impact of anthropogenic CH_4_ on shallow aquifers requires differentiation between natural *in situ* microbial CH_4_ from the migration of microbial or thermogenic CH_4_ from depth, from thermogenic CH_4_ that migrates from an even greater depth due to anthropogenic activities^45^. Although water samples commonly collected from domestic or farm water wells are used to define baseline conditions^46, 47^, Jackson and Heagle^48^ state that such receptor monitoring does not yield baseline concentrations of the aquifer or the regional groundwater supply. We suggest that not wells, but high-resolution profiling be used to provide the much-needed information in environmentally sensitive aquifer systems. This profiling only needs to extend deep enough below the aquifer to allow its evolution to and in the aquifer to be characterized. Based on the profile development in the current study, we suggest the profiling terminate 20–50 m below the bottom of the aquifer. We also suggest, based on the profiles generated in the current study, the sample interval could be reduced from 3m in the current study to 5 m. Assuming the base of the aquifer is located about 50 m BG (a common aquifer depth reported)^47^, and the core hole is terminated 50 m below the base of the aquifer, 20 samples would be collected for analysis. This approach can be integrated with existing drilling programs for new assets (production wells, observation wells, groundwater wells, etc.), where the additional geochemical costs are often negligible compared to drilling costs that could be offset by the potential for major reductions in the future costs associated with fugitive gas migration source tracking and potable aquifer impact assessments.

## Methods

### Drilling, Sampling, and Analyses

Rotary drilling and geologic logging were performed between September 2013 and September 2014 to 150, 200, 1502, and 1432 m below ground surface (BG) at Sites 2, 5, 6, and 7, respectively (Sites 6 and 7 were located ~800 m apart) (Fig. 1). Continuous core sampling was conducted at all four sites and mud gas logging at Sites 6 and 7 only.

Core samples (~100–150 mm long) were collected from Sites 2 and 5 at 3 m intervals and analyzed for: (1) wet and dry densities (*ρ*_*m*_ and *ρ*_*d*_) and gravimetric water contents (*w*) (used to calculate total porosity, *n*_*T*_); (2) dissolved anion chemistry (porewater was squeezed from core samples); and (3) CH_4_, ethane (C_2_H_6_), and propane (C_3_H_8_) concentrations (ppmV). Gas concentrations were measured in the headspace of Isojars^®^ flushed with inert N_2_ or Ar prior to sealing (~440 cm^3^). Core samples collected in Isojars^®^ at Sites 2 and 5 were allowed to equilibrate with the headspace at standard temperature (25 °C) and pressure (1 atm) for 30 and 101 d, respectively, prior to analyses. For analyses, about 10 cm^3^ gas from the headspace was collected from each Isojar^®^ with a 60 cm^3^ syringe and injected into the septa port of an Agilent 7890 gas chromatograph (GC) equipped with a flame ionization detector (FID) to measure light hydrocarbons and a thermal conductivity detector to measure O_2_ and N_2_. Based on analyses conducted on Scotty^TM^ 17 L calibration gas standards (concentrations of 0.0010, 0.01, 0.1, and 10% CH_4_ and 0.0010, 0.01, 0.1, and 1.0% C_2_H_6_ and C_3_H_8_) and on replicate core samples, the accuracy of the analytical method was ± 5%. The limit of detection (LOD) and limit of quantification (LOQ) for CH_4_, C_2_H_6_, and C_3_H_8_ were determined to be 1.1 and 11 ppm, respectively. Concentrations measured in the headspace of the Isojars^®^ were converted to dissolved concentrations (mg L^−1^) using a previously outlined method^33, 49^. These calculations used the core-derived n_T_ values.

At Site 2, the mud gas collection cylinder, located in the mud tank, was connected to an Agilent 490 gas chromatograph (GC) and an IsoTube^®^ gas-sampling manifold. A Los Gatos Research Inc. (LGR) methane carbon isotope analyzer (MCIA)-24-EP CH_4_ was connected in line with the outlet of the IsoTube^®^ gas sampler at Sites 5 and 6. The C_1_-C_5_ concentrations were measured on the mud gas using the Agilent GC every 90 s with a repeatability of 0.5% at 1 mol% and an LOD of 1 ppmV. The LGR CH_4_ analyzer measured CH_4_ concentrations (0–4,000 ppmV) and δ^13^C-CH_4_ values over a CH_4_ concentration range of 500–10,000 ppmV every 50 s. The analytical error for the δ^13^C-CH_4_ values was ± 1.0‰, based on LGR’s model specifications; however, field conditions may have raised the analytical error to ± 1.0‰ - 2‰. Although the LGR CH_4_ analyzer required no internal calibration during sampling, a CH_4_ calibration standard (CH_4_ = 10,000 ppmV, δ^13^C-CH_4_ = −42.3‰) was analyzed every 6 h as a data quality assurance measure to verify instrument accuracy and precision. The δ^13^C-CH_4_ values reported from the LGR were confirmed by submitting 10 gas samples covering the range in values (−67 to −80‰) measured in the Isojars^®^ and analyzed on the LGR at Site 5 to the Isotope Science Lab, University of Calgary. These duplicate results were in good agreement (mean difference = 0.15 ± 0.7‰). δ^13^C analyses were reported in permil (‰) relative to Vienna Pee Dee Belemnite (VPDB), with an accuracy and precision better than ± 0.2‰.

The CH_4_ concentrations from mud gas profiling at Sites 2 and 5 yielded the same depth trend as the core-jar samples but were lower; this was attributed to dilution by drilling fluid during hole advancement followed by dilution with atmospheric gases during sample collection in the mud tank^32^. The strong linear relationship (y = 13.6x + 65.1; R^2^ = 0.83, n = 76) between the dissolved CH_4_ concentrations (mg L^−1^) from core samples collected at Sites 2 and 5 vs. CH_4_ concentrations measured during mud gas logging (ppmV) was used to convert CH_4_ concentrations from mud gas to dissolved gas concentrations at Sites 2 and 5. Because the collection cylinder used to capture mud gas at Site 6 was reconfigured (to minimize atmospheric contamination into the mud gas line) from that used at Sites 2 and 5, calculated dissolved CH_4_ concentrations at Site 6 were determined by multiplying the equation to convert CH_4_ concentrations from mud gas to dissolved gas concentrations at Sites 2 and 5 (y = 13.6x + 65.1) by 0.25 to account for dilution of the mud gas concentrations during hole advancement. The strong linear relationship (y = 0.05x–17.6; R^2^ = 0.88, n = 56) between dissolved CH_4_ concentrations (mg L^−1^) vs. Cl^−^ concentrations (mg L^−1^) measured from core samples collected at Sites 2 and 5 was used to convert calibrated dissolved CH_4_ concentrations (mg L^−1^) collected during mud gas logging at Site 6 to dissolved Cl^−^ concentrations.

IsoTubes^®^ collected at Site 2 were shipped to the G.G. Hatch Stable Isotope Laboratory at the University of Ottawa, Canada, where they were stored for 480 d prior to analysis of δ^13^C-CH_4_ using a Thermo Finnigan Delta^Plus^Advantage IRMS coupled with a VarioEL III, with an accuracy and precision better than ± 0.2‰. IsoTube^®^ samples collected from Site 7 were shipped to Isobrine Solutions Inc., where they were analyzed within 60 days on a Thermo Finnigan MAT252 IRMS, with an accuracy and precision better than ± 0.5‰. Real-time mud gas δ^13^C-CH_4_ values were measured using the LGR CH_4_ and δ^13^C analyzer at Sites 5 and 6. A slight enrichment in δ^13^C-CH_4_ values measured on Isotube^®^ samples from Site 7 compared to values measured during mud gas isotope logging at Site 6 (+2.9‰ ± 1.6‰; n = 25; Fig. 4) is attributed to experimental error and/or minor secondary microbial reactions (e.g. methane oxidation) during sampling and storage of the Isotube^®^ and core samples^33^. To test the reliability of the tubes analysis 32 tubes collected at Sites 2, 3, and 4 that were initially run at 480 d after sampling were resubmitted to the University of Ottawa for re-analysis of CH_4_ and δ^13^C-CH_4_ 1160 d after collection. Results showed that over the additional ~2.5 a of storage since the initial analysis (480 d), the concentration of CH_4_ decreased by an average of 13.5% (mean = 13.5 ± 7.0 ppm; n = 32) and the δ^13^C-CH_4_ shifted by an average of 0.87‰ (mean = 0.87 ± 1.44‰, n = 32). These data show that long-term storage in Isotubes have a minimal impact on the CH_4_ concentrations and δ^13^C-CH_4_ values.

Additional details on the methods and measured values from Sites 2 and 5 are provided elsewhere^16, 33^.

### Transport Modeling

Simulation of the diffusive transport of the dissolved CH_4_, Cl^−^ and δ^13^C-CH_4_ profiles was based on modelling conducted^16^ on the shallow (<165 m BTS) profiles at Site 2. The simulations were performed using a commercially available finite element model (CTRAN/W)^50^. In the current model, we incorporated the deeper profiles through the Pierre Shale Fm obtained from Sites 6 and 7 and the shallow datasets from Sites 2 and 5 (not modeled in ref. 16). The objectives of the modeling were to assess whether the measured profiles can be attributed to diffusion-dominated solute transport and, if so, can the CH_4_, Cl^−^, and δ^13^C-CH_4_ profiles from each site be explained based on our current understanding of the glacial history of the region (Table S1).

The simulations of CH_4_, Cl^−^, ^12^C-CH_4_, and ^13^C-CH_4_ transport were based on transient one-dimensional diffusive transport:

1 $${n}_{e}\frac{\partial C}{\partial t}={n}_{e}{D}_{e}\frac{{\partial }^{2}C}{\partial {z}^{2}},$$

where *n*_*e*_ is the effective porosity, *D*_*e*_ is the effective diffusion coefficient (m^2^ a^−1^), *C* is the mass concentration of the solute (g m^−3^), *z* is distance (m), and t is time (a). *D*_*e*_ in equation (1) was defined according Fick’s first law as:

2 $${J}_{d}=-{n}_{e}{D}_{e}\frac{\partial C}{\partial z},$$

where *J*_*d*_ is the diffusive mass flux rate (g m^−2^ s^−1^).

The diffusive transport of ^12^C-CH_4_ and ^13^C-CH_4_ were determined separately and used to calculate the δ^13^C-CH_4_ profile based on Eqn. 3 ^40^:

3 $${\delta }^{13}C-C{H}_{4}=\frac{{R}_{({}^{13}C-C{H}_{4})}}{{R}_{std}}-1\,,$$

where ${R}_{({}^{13}C-C{H}_{4})}$ is the isotope ratio of CH_4_, defined as

4 $${R}_{({}^{13}C-C{H}_{4})}=\frac{\frac{c({}^{13}C-C{H}_{4})}{{M}_{{}^{13}C{H}_{4}}}}{c({}^{12}C-C{H}_{4})/{M}_{{}^{12}C{H}_{4}}},$$

where *R*_*std*_ is the standard isotopic ratio for Pee Dee Belemnite (PDB; 0.011237) and *M* is the molecular mass.

The values of *D*_*e*_ for Cl^−^ were assumed to be 2.1 × 10^−10^ and 2.3 × 10^−10^ m^2^ s^−1^ in the till^28^ and Pierre shale^23^, respectively. Values of *D*_*e*_ for CH_4_ were assumed to be 2.4 × 10^−10^ m^2^ s^−1^ for both the till and shale^51^. The *n*_*T*_ values were determined on individual core samples. Mean values of *n*_*T*_ for the till and shale were 33.2 ± 4.4 and 34.3 ± 2.9, respectively, were used in the modelling. The value of *n*_*e*_ for CH_4_ was assumed to be equal to the total porosity (*n*_*T*_)^51^. The *n*_*e*_ values for Cl^−^ were determined to be *n*_*e*_ = *n*_*T*_ for the till and *n*_*e*_ = 0.7*n*_*T*_ for the Pierre Shale Fm^16^. The value of *D*_*e*_ for ^12^C-CH_4_ was assumed to be the same as that for CH_4_ transport in the shale (2.4 × 10^−10^ m^2^ s^−1^). The difference in molecular mass between ^12^CH_4_ and ^13^CH_4_ results in the *D*_*e*_ for ^12^CH_4_ exceeding that of ^13^CH_4_ by a factor of 1.0195 based on measurements of diffusion in air^52^. This ratio was assumed to apply to our simulations for aqueous diffusion. As such, the value of *D*_*e*_ for ^13^C-CH_4_ was 2.35 × 10^−10^ m^2^ s^−1^.

One-dimensional transport modelling was undertaken in two phases. Phase 1 defined the distributions of CH_4_, Cl^−^, and δ^13^C-CH_4_ across the shale prior to glaciation, and Phase 2 assessed the impact of Sutherland and Saskatoon Gr glacial periods on the final (present-day) profiles for CH_4_, Cl^−^, and δ^13^C-CH_4_ at Sites 2 and 5. Details of the methodologies used in each phase of modelling are presented in Supplemental Information.

The relative root mean square error (RRSME)^53^ was used to compare the simulated and measured concentration profiles for each scenario and determine the best-fit model results (data not presented).

## Electronic supplementary material

Supplemental Information
