# Supplementary material for: Fate and Transport of Shale-derived, Biogenic Methane
Source: Sci Rep. 2017 Jul 7;7:4881. doi: 10.1038/s41598-017-05103-8 (PMC5501783; doi:10.1038/s41598-017-05103-8)
Supplement: Supplementary file 1 — Supplemental Information [file 41598_2017_5103_MOESM1_ESM.pdf]

Supporting Information for

[Fate and Transport of Shale-derived, Biogenic Methane]

[M. Jim Hendry[1*], Erin. E. Schmeling[1], S. Lee Barbour[2], M. Huang[2] and Scott O.C. Mundle[3]]

[[1]*Department of Geological Sciences, University of Saskatchewan, 114 Science Place, Saskatoon, SK, Canada S7N 5E2.*

[2]*Department of Civil and Geological Engineering, University of Saskatchewan, 57 Campus Dr. Saskatoon, SK, Canada S7N 5A9.*

[3]*Great Lakes Institute for Environmental Research, University of Windsor, 401 Sunset Ave., Windsor, ON, Canada N9B 3P4.*]

- *corresponding author* jim.hendry@usask.ca

Methods: Details on Transport Modelling of CH_4, Cl^-, and $\delta^{13}C$-CH_4

One-dimensional ransport modelling was undertaken in two phases. Phase 1 defined the distributions of CH_4, Cl^-, and $\delta^{13}C$-CH_4 across the shale prior to glaciation, and Phase 2 assessed the impact of Sutherland and Saskatoon Gr glacial periods on the final (present-day) profiles for CH_4, Cl^-, and $\delta^{13}C$-CH_4 at Sites 2 and 5.

Phase 1: Defining Pre-Glacial Profiles

The initial conditions for the model were simulated distributions of CH_4, Cl^-, and $\delta^{13}C$-CH_4 across the shale prior to glaciation. We estimated the upper and lower boundary conditions at the end of the Cretaceous based on the near linear CH_4, Cl^-, ^{12}C-CH_4, and ^{13}C-CH_4 profiles in the Pierre Shale Fm (between 600 and 0 m BTS) at Sites 6 and 7. In the case of the CH_4 and Cl^- profiles, the lower and upper boundaries were held constant at 280 and 180 g m^{-3} and 6200 and 4000 g m^{-3}, respectively. The lower and upper boundary conditions for ^{12}C-CH_4 and ^{13}C-CH_4 were set at 276.9 and 3.1 g m^{-3} (-69.5‰ for $\delta^{13}C$-CH_4) and 0 and 0 g m^{-3}, respectively. The initial CH_4 and Cl^- concentrations were assigned concentrations of 0 and 19,400 g m^{-3} (roughly sea water) and the initial concentrations for both stable isotopes of C were set at 0 g m^{-3}. The three models were run for 72 Ma (age of the Pierre Shale Fm) and resulted in near steady-state concentrations throughout the Pierre Shale Fm prior to glaciation.

Phase 2: Defining Glacial Profiles

The impact of Sutherland and Saskatoon Gr glacial periods on the final (present-day) profiles for CH_4, Cl^-, and $\delta^{13}C$-CH_4 at both Sites 2 and 5 was assessed by simulating nine scenarios that represented the probable ranges in depositional times for the Sutherland Gr (*i.e.,* 0.6, 1.0, and 1.6 Ma) and Saskatoon Gr (*i.e.,* 0.1, 0.3, and 0.6 Ma) tills on CH_4, Cl^-, and $\delta^{13}C$-CH_4 profiles. Based on our geologic logs, the measured thicknesses of the tills (9 m of

Sutherland Gr till and 45 m of Saskatoon Gr till) were deposited in two sequential glacial periods at Site 5. Due to the lack of Sutherland Gr till at Site 2, we assumed that 9 m of Sutherland Gr till (*i.e.,* the same thickness as the Sutherland Gr till at Site 5) was initially deposited but completely eroded prior to deposition of 36 m of Saskatoon Gr till.

The impact of glacial deposition on the upper boundary concentrations over geologic time is unknown. As a result, we were required to make several simplifying assumptions. The upper boundaries for both CH_4 and Cl^- at Site 2 were located at the top of the Pierre Shale Fm based on the present-day CH_4 and Cl^- concentration profiles across the till-shale interface. The CH_4 and Cl^- concentrations were assigned values of 95 and 2,200 g m^{-3}, respectively, at the onset of the Sutherland Gr and 6.8 and 240 g m^{-3} at the onset of the Saskatoon Gr. The upper boundary concentrations at the time of the deposition of the Saskatoon Gr till were based on current measurements; however, because of a lack of information, the Cl^- concentration at the time of Sutherland Gr till deposition was determined based on the quality of the fit to the observed data in the subsequent phases of the simulation and the CH_4 concentration calculated using the linear equation between Cl^- and CH_4. At Site 5, the upper boundary conditions for CH_4 and Cl^- at the time of deposition of the Sutherland Gr till was fixed at 95 and 2200 g m^{-3}, respectively, consistent with Site 2. The simulated profiles at the end of Sutherland Gr till deposition period were used to define the initial profile for the simulation of Saskatoon Gr till deposition. The upper boundary conditions for CH_4 and Cl^- were fixed at 3.5 and 66 g m^{-3} during Saskatoon Gr till deposition (based on present-day measurements). Based on their present-day elevations of Sutherland and Saskatoon Gr tills, the upper boundary conditions for CH_4 and Cl^- at Site 5 were set at 45 and 13 m BG during the deposition of Sutherland and Saskatoon Gr tills, respectively.

The initial concentrations in both tills were set at 0 g m^{-3} for both CH_4 and Cl^-. These assumptions are supported by low background concentrations of Cl^- and CH_4 (about 20 and 0 g m^{-3}, respectively) measured in thick, recently deposited (Battleford Fm; Table S1) till[17,48] and believed to reflect the concentrations in tills immediately after till deposition[16].

In the case of $\delta^{13}C$-CH_4, the initial $\delta^{13}C$-CH_4 profile was assumed to be constant at -69.5‰ prior to glaciation, consistent with the presence of near steady-state CH_4 diffusion and the underlying $\delta^{13}C$-CH_4 boundary. Constant concentrations of 93.993 g m^{-3} for ^{12}C-CH_4 and 1.007 g m^{-3} for ^{13}C-CH_4 (a $\delta^{13}C$-CH_4 of -102.5‰) were set as upper boundary conditions at the top of the Sutherland Gr till during the deposition of the Sutherland till at Sites 2 and 5. The simulated profiles of ^{12}C- and ^{13}C-CH_4 at the end of Sutherland Gr till deposition were used as initial concentrations to simulate Saskatoon Gr till deposition. Based on present-day measurements of the total CH_4 concentration and the value of $\delta^{13}C$-CH_4 at the top of Saskatoon till for both sites, upper boundary concentrations of ^{12}C- and ^{13}C-CH_4 assigned to the top of Saskatoon till were 6.68 and 0.07 g m^{-3} (a $\delta^{13}C$-CH_4 of -85‰) at Site 2 and 3.46 and 0.04 g m^{-3} (a $\delta^{13}C$-CH_4 of -85‰) at Site 5, respectively. The concentrations for total CH_4 used in the simulations were those used in the CH_4 simulations.

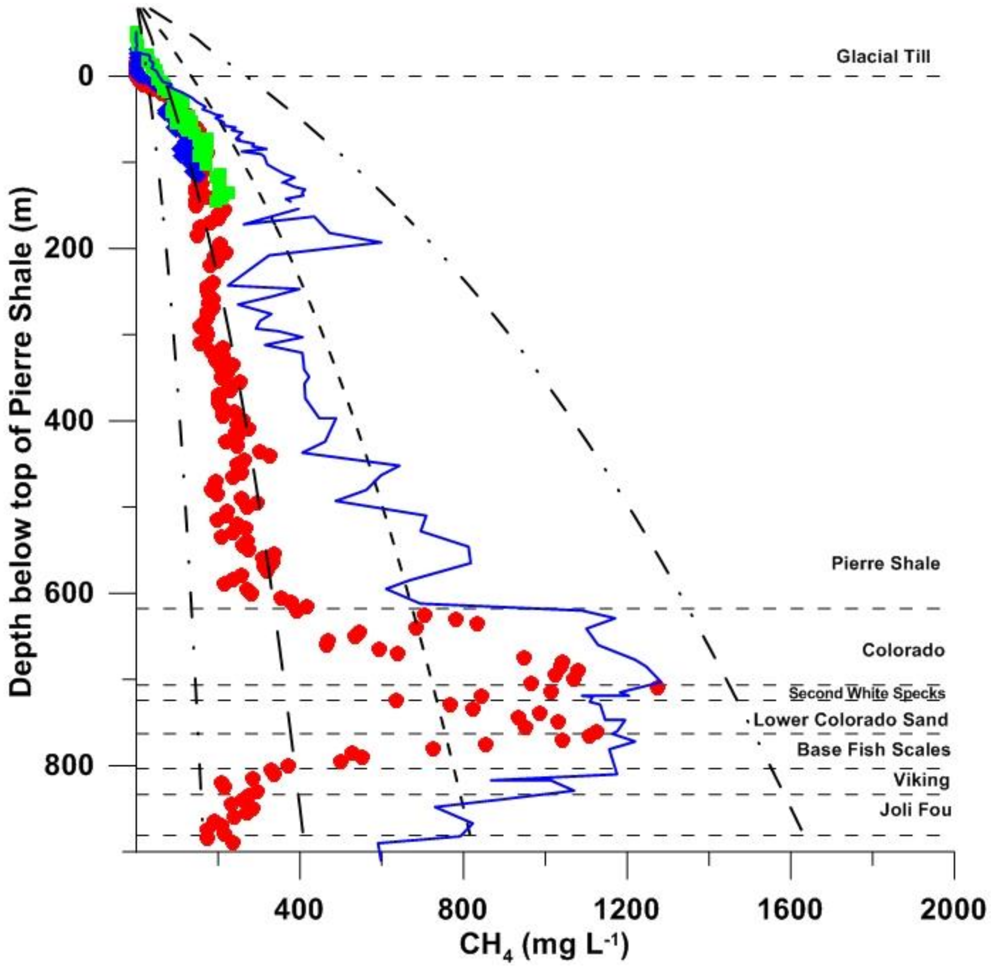

Fig. S1 Calculated CH_4 concentrations through the Quaternary deposits and Cretaceous shales from Isojars® at Sites 2 (solid blue diamonds) and 5 (solid green squares) and mud gas logs at Site 6 (solid red circles). The estimated CH_4 solubility profile based on Site 5 Isojars® and cuttings samples collected in IsoJars® at Site 7 is represented by the solid blue line based on *in vitro* gas concentrations corrected for *in situ* temperatures and pressures. Dashed lines represent calculated CH_4 gas solubility profiles based on gas concentrations of 10, 25, 50, and 100% CH_4 (left to right). The CH_4 gas solubilities were corrected for *in situ* temperature and pressure.

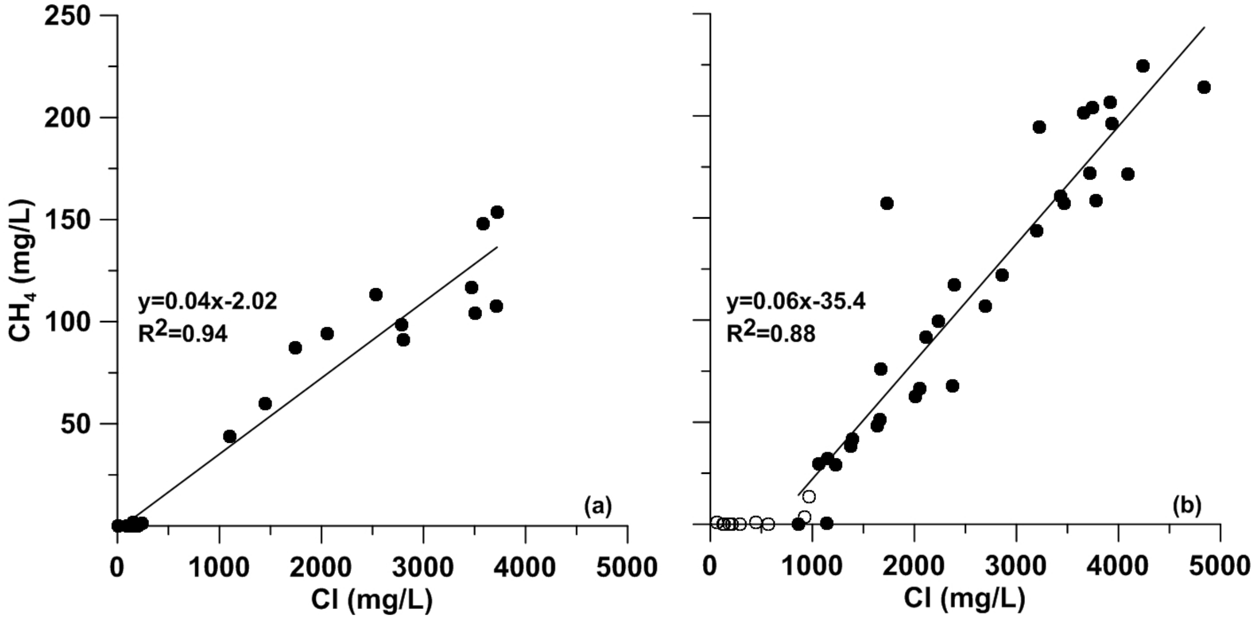

Fig S2. Cross plots of dissolved CH_4 versus Cl^- from core samples at Sites 2 and 5 are presented in (a) and (b), respectively. The open symbols in (b) are from the upper boundary where CH_4 is oxidized while Cl^- continues to diffuse. The best fit liner correlation and associated R^2 values for all data points are presented for each site.

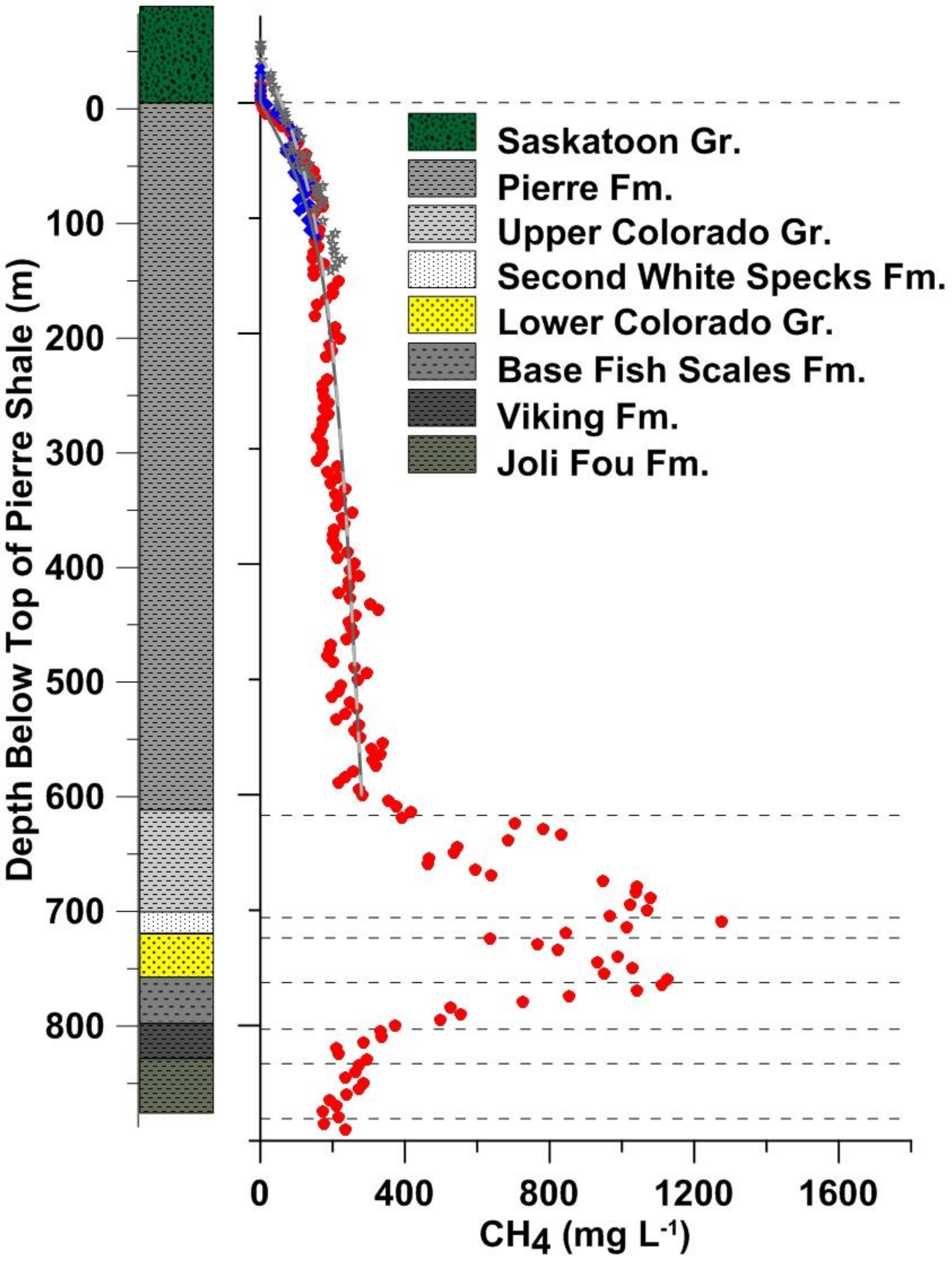

Figure S3. Geology and dissolved CH_4 concentrations through the Quaternary deposits and Cretaceous shales at Sites 2, 5, 6, and 7. Isojar® data from Sites 2 and 5 are shown as solid blue diamonds and open grey stars, respectively, and calibrated mud gas data from Sites 2, 5, and 6 as dashed black line, solid black line, and solid red circles, respectively.. The best-fit 1-D diffusive modeling results (1.6 Ma and 0.3 Ma for Sutherland and Saskatoon Group tills, respectively) are presented as solid lines (Sites 2 and 6) and dashed lines (Site 5).

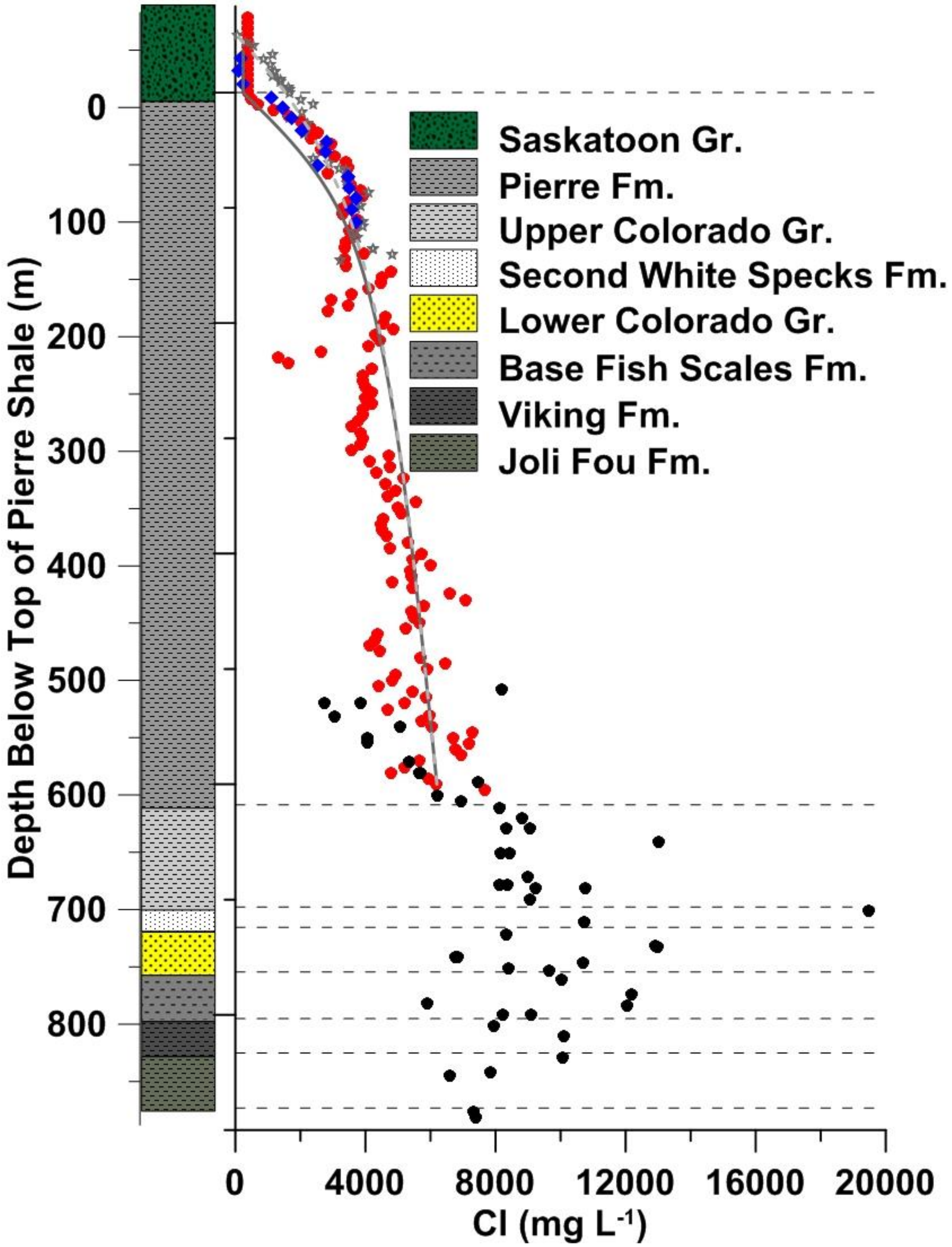

Figure S4. Geology and Cl^- concentrations through the Quaternary deposits and Cretaceous shales at Sites 2, 5, 6, and 7. Squeezed core data from Sites 2 and 5 are shown as solid blue diamonds and open grey stars, respectively, and calibrated mud gas data from Sites 2, 5, and 6 as dashed black line, solid black line, and solid red circles, respectively. Measured Cl^- concentrations from the base of the Pierre Fm to the Joli Fou Fm from a drill site located 250 km SE of the study area[22] are presented as solid black circles. respectively. The best-fit 1-D diffusive modeling results (1.6 Ma and 0.3 Ma for Sutherland and Saskatoon Group tills, respectively) are presented as solid lines (Sites 2 and 6) and dashed lines (Site 5).

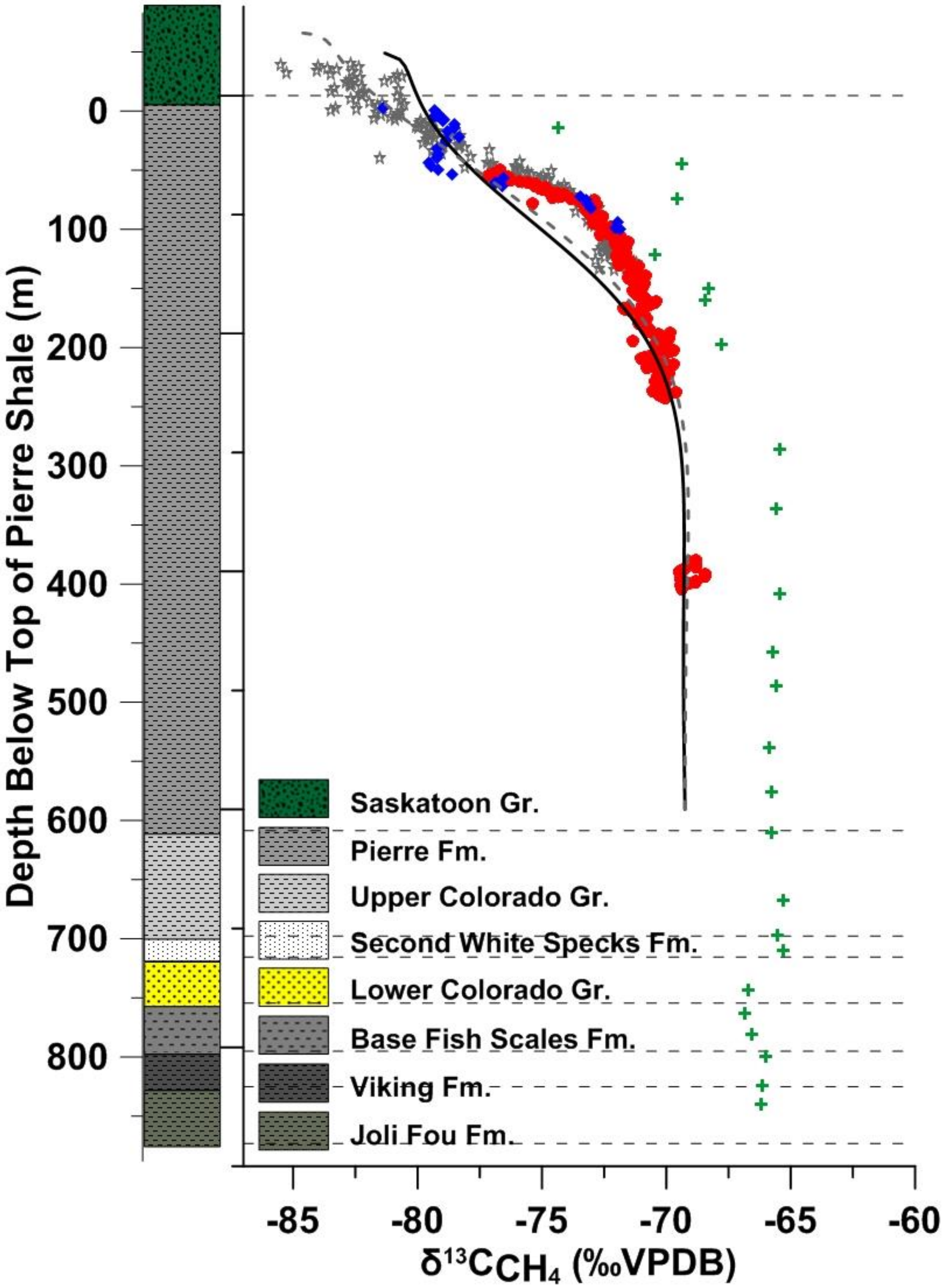

Figure S5. Geology and $\delta^{13}C$-CH_4 values through the Quaternary deposits and Cretaceous shales at Sites 2, 5, 6, and 7. IsoTube® data from Sites 2 and 7 are presented as solid blue diamonds and green crosses, respectively, and mud gas data from Sites 5 and 6 as open grey stars and solid red circles, respectively. The best-fit 1-D diffusive modeling results (1.6 Ma and 0.3 Ma for Sutherland and Saskatoon Group tills, respectively) are presented as solid lines (Sites 2 and 6) and dashed lines (Site 5).

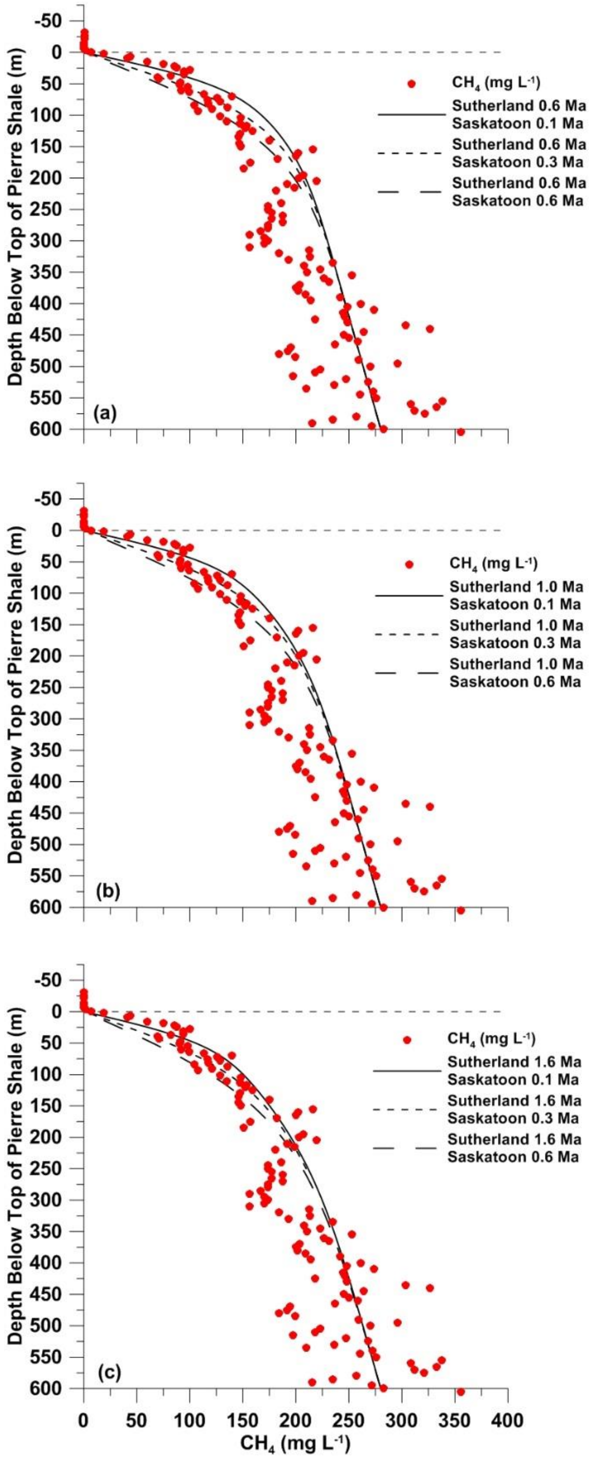

Fig. S6 Simulated and measured CH_4 concentration profiles for Sites 2 and 6 for different timings of the deposition of the Sutherland Group till (0.6 Ma (a), 1.0 Ma (b), and 1.6 Ma (c)) and a range in deposition times of the Saskatoon Group till (0.1, 0.3, and 0.6 Ma).

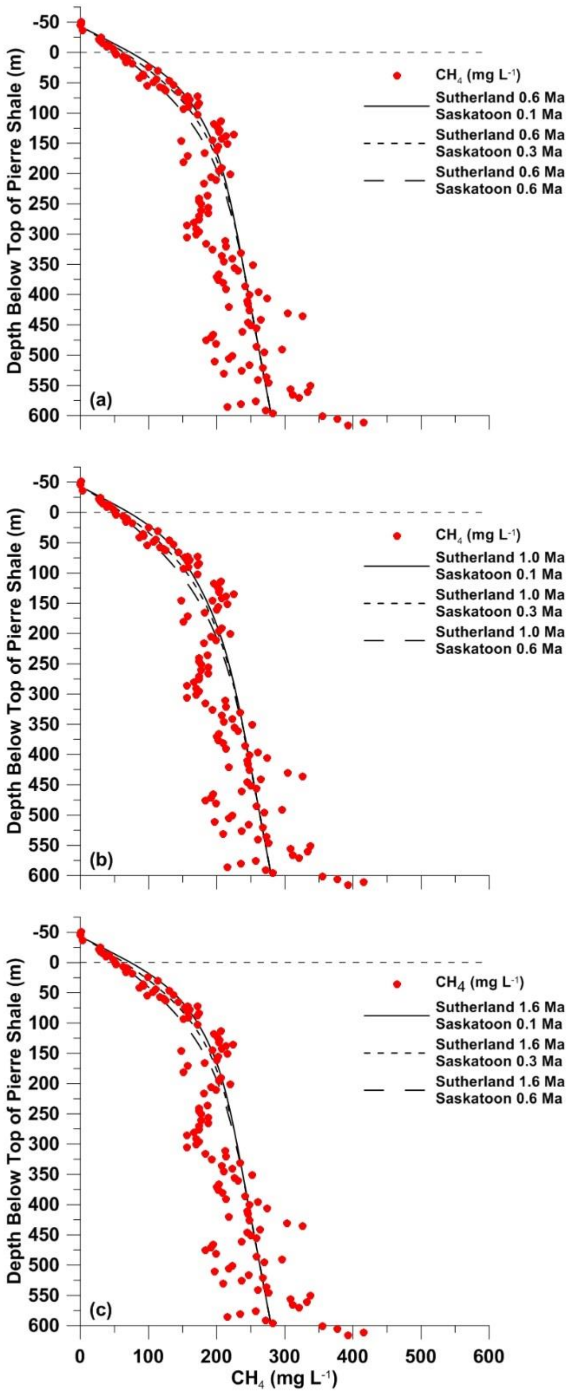

Fig. S7 Simulated and measured CH_4 concentration profiles for Sites 5 and 6 for different timings of the deposition of the Sutherland Group till (0.6 Ma (a), 1.0 Ma (b), and 1.6 Ma (c)) and a range in deposition times of the Saskatoon Group till (0.1, 0.3, and 0.6 Ma).

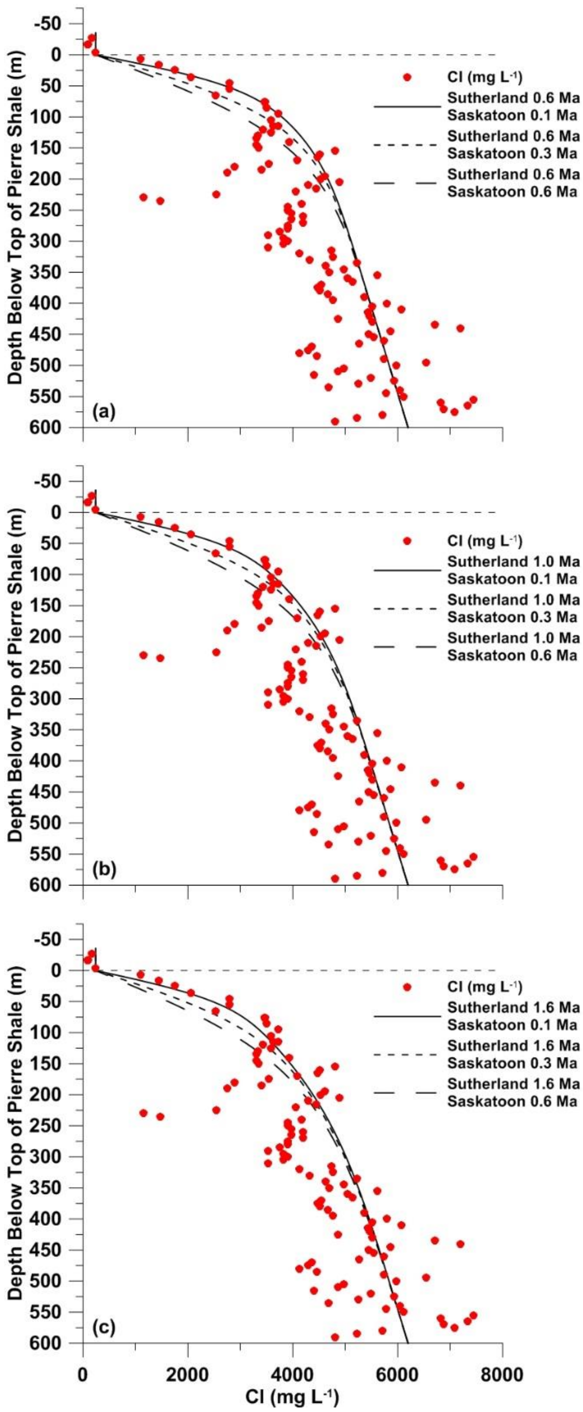

Fig. S8 Simulated and measured Cl^- concentration profiles for Sites 2 and 6 for different timings of the deposition of the Sutherland Group till (0.6 Ma (a), 1.0 Ma (b), and 1.6 Ma (c)) and a range in deposition times of the Saskatoon Group till (0.1, 0.3, and 0.6 Ma).

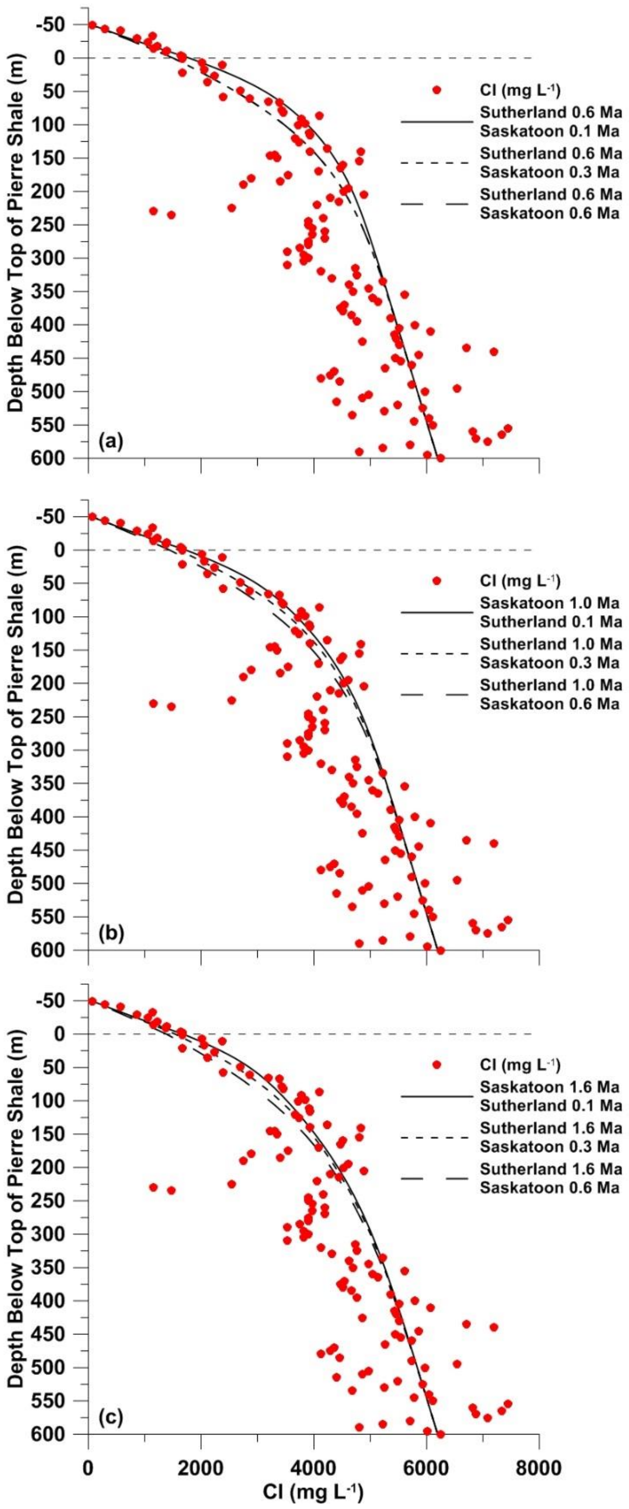

Fig. S9 Simulated and measured Cl^- concentration profiles for Sites 5 and 6 for different timings of the deposition of the Sutherland Group till (0.6 Ma (a), 1.0 Ma (b), and 1.6 Ma (c)) and a range in deposition times of the Saskatoon Group till (0.1, 0.3, and 0.6 Ma).

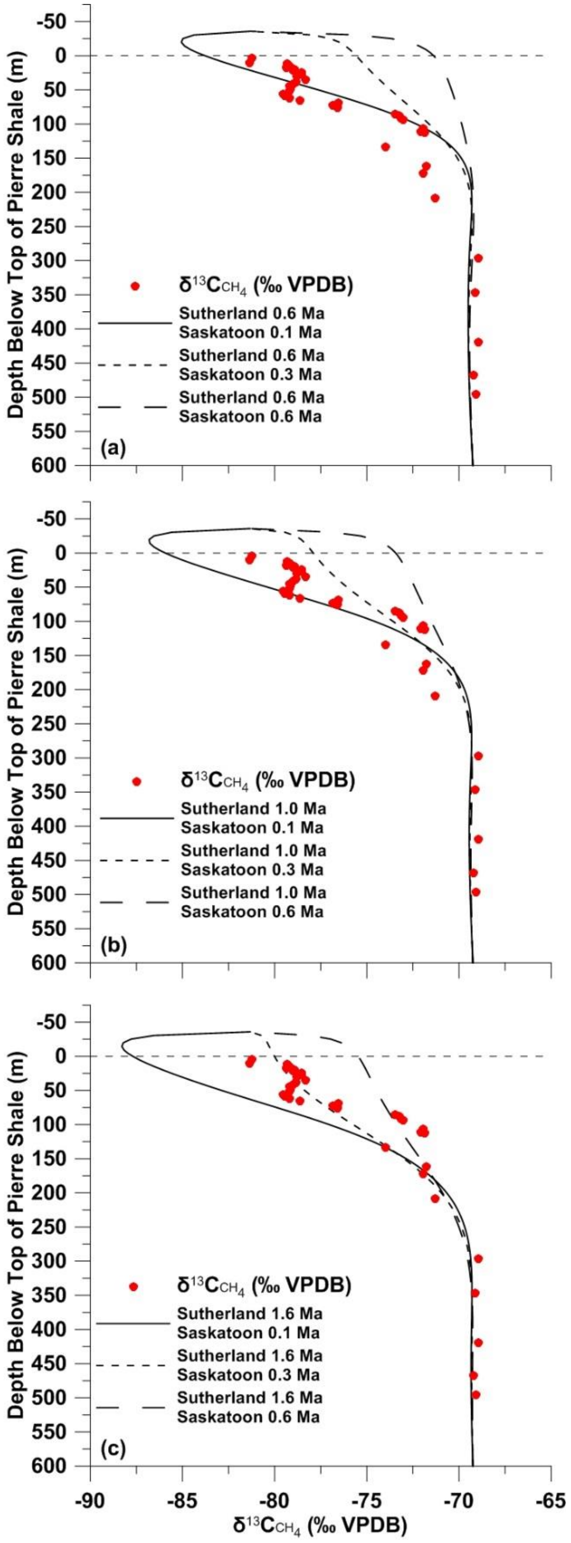

Fig. S10 Simulated and measured $\delta^{13}C$-CH_4 profiles for Sites 2 and 6 for different timings of the deposition of the Sutherland Group till (0.6 Ma (a), 1.0 Ma (b), and 1.6 Ma (c)) and a range in deposition times of the Saskatoon Group till (0.1, 0.3, and 0.6 Ma).

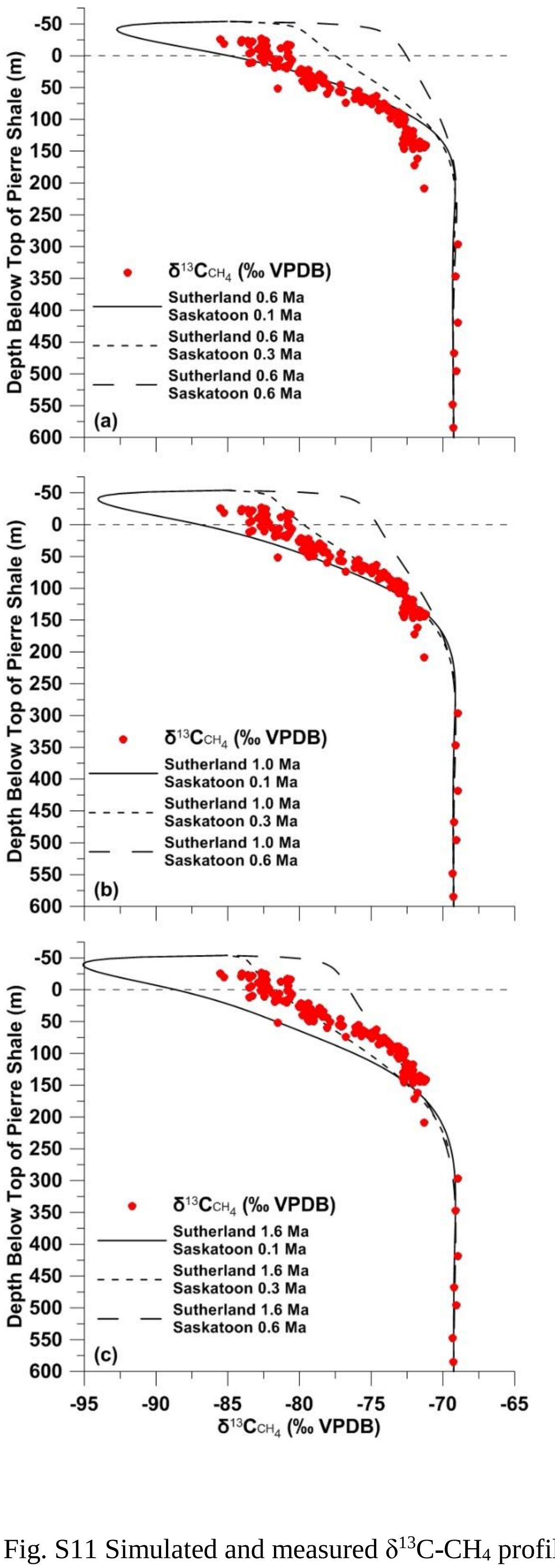

Fig. S11 Simulated and measured $\delta^{13}C$-CH_4 profiles for Sites 5 and 6 for different timings of the deposition of the Sutherland Group till (0.6 Ma (a), 1.0 Ma (b), and 1.6 Ma (c)) and a range in deposition times of the Saskatoon Group till (0.1, 0.3, and 0.6 Ma).

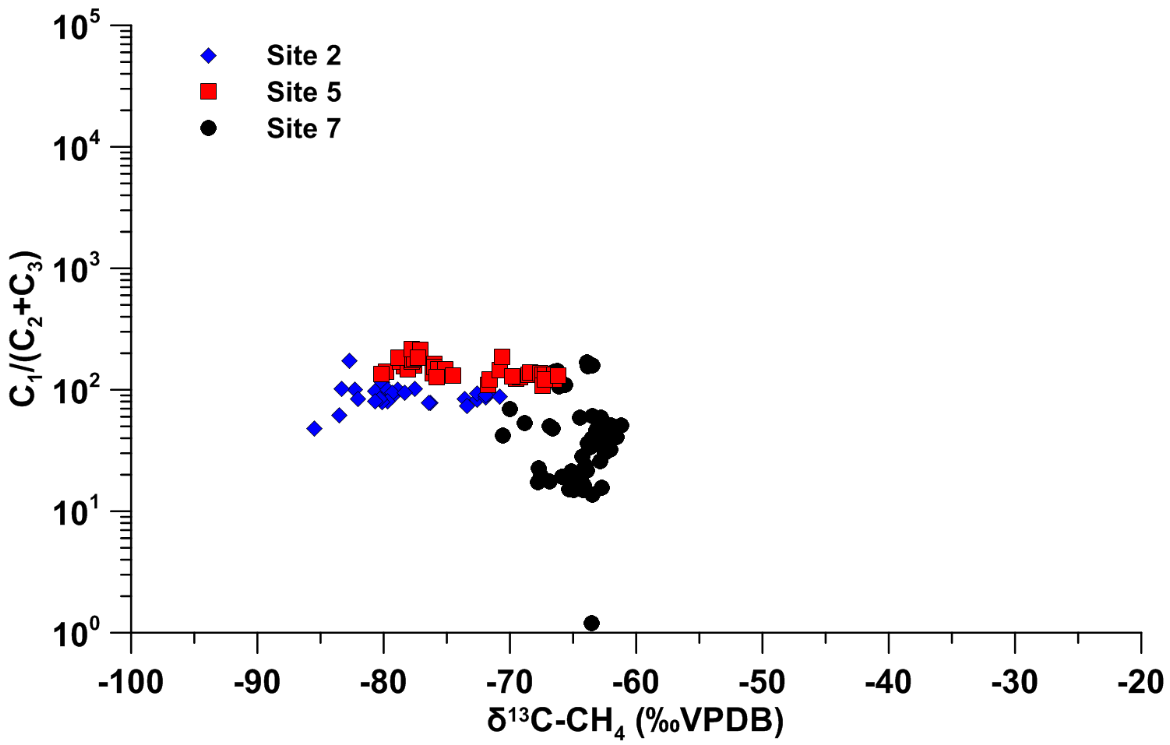

Fig S12. Crossplot of $\delta^{13}C-CH_4$ values vs. C1/(C2+C3) (after Bernard et al., 1978) of gas samples from the glacial tills and Cretaceous shales at Sites 2, 5, and 7.

Table S1. Summary of initial and boundary conditions, present-day till thicknesses, and simulated depositional times for Sutherland (Sut.) and Saskatoon (Sas.) Group tills applied to the Cl^- and CH_4 profiles at Sites 2 and 5.

	Upper BC[#] at the top of Pierre Shale ($mg\ L^{-1}$)		Lower BC (600 m BG[##]) ($mg\ L^{-1}$)	Simulated depositional thicknesses (m)	Initial concentration in deposited tills ($mg\ L^{-1}$)
	Sut.	Sask.			
**Site 2**					
CH_4 model	95	0.3	280	36 (Sas.)	0
Cl^- model	2200	240	6200	36 (Sas.)	0
**Site 5**					
CH_4 model	95	0	280	9+45 (Sut. + Sas.)	0
Cl^- model $^-$	2200	60	6200	9+45 (Sut. + Sas.)	0

[#]BC, boundary condition

[##]BG, below ground surface

References:

Bernard, B. B., Brooks, J. M. & Sackett, W. M. Light hydrocarbons in recent Texas continental shelf and slope sediments. *J. Geophys. Res*. **83**(C8):4053-4061 (1978).
